# Effects of Synchronized Ovulation Protocols on Reproductive Performance of Beef Cattle in Korea: A Retrospective Study of 755 Cases

**DOI:** 10.3390/vetsci12101001

**Published:** 2025-10-16

**Authors:** Jong-Geol Ha, Tae-Gyun Kim, Sung-Ho Kim, Sang-Yup Lee, Saet-Byul Kim, Seung-Joon Kim, Won-Jae Lee

**Affiliations:** 1 College of Veterinary Medicine, Kyungpook National University, Daegu 41566, Republic of Korea; aentifriz@gmail.com (J.-G.H.); xo1621@naver.com (T.-G.K.); ideat12@knu.ac.kr (S.-H.K.); kjoon00@knu.ac.kr (S.-J.K.); 2 Bovivet, Gumi 39133, Republic of Korea; bovivet@naver.com (S.-Y.L.); sbkim@gmail.com (S.-B.K.); 3 Institute for Veterinary Biomedical Science, Kyungpook National University, Daegu 41566, Republic of Korea

**Keywords:** CIDR protocol, GPG protocol, GPPG protocol, Hanwoo cattle, pregnancy rate, timed artificial insemination

## Abstract

Reproductive outcomes following synchronized ovulation protocol in beef cattle are influenced by multiple factors, making protocol selection based on farm-specific conditions essential. This retrospective study analyzed the relationship between pregnancy rates and associated factors under the CIDR (GnRH with CIDR insertion–PGF_2α_ with CIDR removal–GnRH), GPG (GnRH–PGF_2α_–GnRH), and GPPG (GnRH–PGF_2α_–PGF_2α_–GnRH) protocols in Hanwoo cattle. The highest pregnancy rate was observed with the CIDR protocol (58.3%), whereas the GPG protocol yielded the lowest (47.5%). The CIDR protocol demonstrated superior suitability compared with the GPG protocol in first service (61.0% vs. 47.0%) and young breeding cattle (parity: 0–2; 61.6–70.0% vs. 47.5–48.6%). The dominant follicle size strongly associated with pregnancy success was 13–16 mm, and the CIDR protocol induced these follicles more frequently than the GPG protocol (50.2% vs. 35.5%). Although interpretive bias may exist from data collected from pregnant animals only, CIDR protocol significantly increased luteinizing hormone levels compared to GPG. The GPPG protocol produced outcomes that were improved relative to the GPG protocol and statistically comparable to the CIDR protocol. These findings addressed the study’s objective, identifying optimal synchronized ovulation strategies and underscoring reproductive management importance for Korean Hanwoo operations.

## Simple Summary

Cattle farms require efficient breeding methods to increase pregnancy rates, but selection of the most suitable hormone treatment protocol for each farm can be challenging. This study compared three hormone-based synchronized ovulation protocols commonly used in Korean cattle farms to identify which protocol is most effective for particular groups of cows. Pregnancy rates, follicle development, and blood hormone levels were evaluated in 540 cows using three protocols: CIDR, GPG, and GPPG. The CIDR protocol achieved the highest pregnancy rate (58.3%), whereas the GPG protocol showed the lowest (47.5%). The CIDR protocol was particularly effective in younger cows and at the first artificial insemination, reaching up to 70% success in some groups. Medium-sized follicles (13–16 mm) produced the highest pregnancy rates, and the CIDR protocol most consistently induced follicles of this optimal size. Blood tests indicated that the CIDR protocol established a more favorable hormonal environment for successful breeding. These findings provide cattle farmers with practical evidence to guide the selection of effective breeding strategies, ultimately improving farm productivity and reducing breeding costs.

## 1. Introduction

Synchronized ovulation in cattle is defined as the artificial induction of estrus onset and ovulation in a large proportion of females at a predetermined time [1]. Timed artificial insemination (TAI) refers to the insemination of such synchronized female herds without requiring estrus detection [2]. These technologies have continuously advanced over the past decades and are now regarded as fundamental reproductive management tools in modern livestock production systems [2,3]. This biotechnology is considered particularly valuable for large-scale farms because it minimizes human error [1] and management costs, concentrates calving during the most favorable seasons for raising newborns [3], and improves production efficiency by reducing open periods [4,5].

Four main types of hormones are primarily used to regulate synchronized ovulation. Gonadotropin-releasing hormone (GnRH) stimulates the release of follicle-stimulating hormone (FSH) and luteinizing hormone (LH) from the anterior pituitary gland, inducing ovulation or atresia of the dominant follicle (DF) and triggering the emergence of new follicular waves [6,7,8]. Prostaglandin F_2α_ (PGF_2α_) binds to specific receptors on luteal cells, causing regression of the corpus luteum (CL) and reducing progesterone (P_4_) production, which enables follicular growth and resumption of estrous cycles [9,10]. P_4_ suppresses estrus expression through negative feedback on GnRH and blocks LH surges to maintain the luteal phase, most commonly delivered via intravaginal progesterone-releasing devices (CIDR) [11,12]. Estradiol (E_2_), when combined with CIDR, resets follicular growth stages and enhances TAI success rates through consistent induction of estrus [4].

Various synchronized ovulation techniques have been developed and applied by exploiting the functions of these hormones and the bovine follicular wave cycle. The CO-Synch+CIDR method (designated as the CIDR protocol in this study) supplies exogenous P_4_ for 5–7 days to suppress LH surges, then administers PGF_2_α along with CIDR removal to induce proestrus; this is followed by GnRH-induced ovulation and TAI 48–72 h later [6]. The Ovsynch protocol involves sequential administration of GnRH, PGF_2_α, and GnRH (designated as the GPG protocol in this study), which induces ovulation of the DF, regression of the CL, and ovulation of a new DF, respectively [1]. The CIDR protocol has been reported to improve pregnancy rates from approximately 48% to 59% [6,12]. The GPG protocol typically achieves pregnancy rates of 40–50% and can increase pregnancy rates to over 50% when initiated at optimal points in the estrous cycle or combined with additional synchronization methods, such as Select Synch, which involves estrus detection followed by AI after the GPG protocol [6,13,14]. The modified 5-day Co-Synch protocol (designated as the GPPG protocol in this study) was developed by shortening the conventional 7-day Ovsynch protocol to 5 days and administering two consecutive PGF_2α_ injections on days 5 and 6 (GnRH–PGF_2α_–PGF2_2α_–GnRH). This protocol improves embryo quality by reducing the period of follicular dominance by two days and addresses incomplete regression of newly formed CLs (less than 5 days old) through double PGF_2α_ administration [15].

However, synchronized ovulation protocols show substantial variability in pregnancy rates and efficiency across different geographical regions, management systems, and biological factors; previous studies have demonstrated that pregnancy rates after ovulation-synchronized breeding range from as low as 10% to over 80%, depending on the protocol used [4,7,15]. Ovulation induction in dairy cows also considerably varies according to parity status [8]. Additionally, grazing-based dairy systems exhibit distinct ovarian responses to hormonal protocols compared with confinement-based operations; seasonal or climatic factors such as heat stress substantially influence synchronization success [16]. These wide variations in response patterns highlight the need for continued research to establish synchronized ovulation strategies capable of maintaining consistent pregnancy rates under diverse cattle management environments. There is an urgent need to evaluate the efficacy of synchronization protocols for beef cattle under East Asian production conditions, including Korea. Although extensive studies have been conducted on Holstein dairy cattle [4,7,9,10,11,14] and foreign beef breeds [2,5,6,12], the reproductive responses of Korean native beef cattle (Hanwoo, *Bos taurus coreanae*) remain insufficiently characterized, especially considering their unique genetic background and adaptation to Korean climatic conditions. This retrospective study aimed to evaluate and compare the reproductive effectiveness of three synchronized ovulation protocols: CIDR and GPG (the most widely utilized methods in Korea) and GPPG protocols in Hanwoo cattle. The specific objectives were to: (1) compare pregnancy rates among protocols, (2) analyze the influence of dominant follicle size and parity on reproductive outcomes, (3) assess protocol performance across service attempts, and (4) characterize hormonal profiles (P_4_, E_2_, and LH) associated with each synchronization method to identify optimal strategies for Korean beef cattle operations. The findings from this retrospective analysis will provide evidence-based guidance to Korean veterinarians and beef cattle producers for selecting optimal synchronized ovulation protocols, ultimately contributing to improved reproductive efficiency and economic sustainability in the domestic Hanwoo industry.

## 2. Materials and Methods

### 2.1. Animal Ethics

This study retrospectively analyzed records collected during routine veterinary herd health management, without introducing procedures beyond normal clinical practices. Therefore, separate ethical committee approval was not necessary. Nonetheless, the research was conducted in full compliance with recognized veterinary ethical principles and professional codes of practice.

### 2.2. Study Location and Animals

Medical records (from 25 April 2024 to 26 January 2025) from three specialized bovine veterinary hospitals located near Daegu, Republic of Korea (latitude: 35°52′ N, longitude: 128°36′ E), were retrospectively analyzed. The inclusion criteria for selecting subjects in this retrospective study based on these medical records were as follows: (1) breeding farms where Hanwoo (Korean native beef cattle) were fed grass silage and concentrate twice daily, with mineral blocks and water provided *ad libitum*; (2) farms where breeding is primarily performed by AI; (3) farms in which herds exist that have been subjected to all three synchronized ovulation protocols (CIDR, GPG, and GPPG); (4) animals that underwent TAI following synchronized ovulation; (5) medical records of heifers at approximately 15 months of age and parous cows at around 50 days postpartum, none of which were suckling their calves; (6) confirmation of ovarian cyclicity prior to synchronized ovulation through transrectal ultrasonography using a 7.5 MHz linear transducer (Easy-Scan Go, IMV, Bellshill, UK), where ovarian cyclicity was defined as the regular and repeated pattern of follicular development, ovulation, and CL formation and regression underlying the reproductive cycle; (7) body condition score (BCS) of 2.0 or higher on a scale from 1 (emaciated) to 5 (obese) to exclude nutritional deficiency factors that could influence pregnancy outcomes; (8) Cows for which the diameter of the DF was measured by ultrasonography on the ovary expected to ovulate on the day of AI. In Korea, to maximize the pregnancy rate after AI, semen is routinely deposited into the uterine horn ipsilateral to the ovary where ovulation is expected in ovulation-synchronized cows, and pre-insemination ultrasonographic examination of the ovarian status is generally performed for this purpose; (9) animals that underwent blood collection on the day of AI for the national livestock disease control programs (including brucellosis testing) under the ‘Guidelines for Livestock Disease Control Project Implementation’ established by the Ministry of Agriculture, Food and Rural Affairs of the Republic of Korea, and tested negative for related diseases; (10) animals that maintained pregnancy as confirmed by ultrasonographic examination of the uterus at approximately 30 and 75 days post-TAI, with cases showing embryonic loss during the second monitoring excluded from the analysis.

All procedures were performed by three cattle specialist veterinarians, each with more than five years of clinical experience and who have performed over 5000 artificial inseminations in cattle. Finally, the results of 755 TAIs after synchronized ovulation in a total of 540 cattle from 12 farms (425 successful pregnancies and 330 failed pregnancies) were retrospectively analyzed. Their BCS averaged 3.3 ± 0.5 (mean ± standard deviation), allowing exclusion of nutritional factors affecting pregnancy rates. The experimental group of 540 cows consisted of nulliparous heifers (parity 0; *n* = 236), young cows (parity 1–2; *n* = 173), mature cows (parity 3–5; *n* = 101), and older cows (parity ≥ 6; *n* = 30). The 755 estrus induction treatments included the CIDR protocol (*n* = 295), GPG protocol (*n* = 124), GPPG protocol (*n* = 305), and natural estrus (*n* = 31).

### 2.3. Synchronized Ovulation Protocols and TAI Procedures

**Figure 1:**
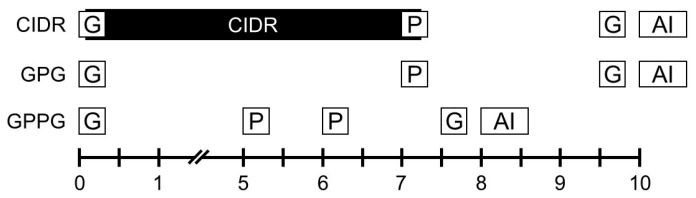
Representative diagram of the three synchronized ovulation protocols evaluated in this retrospective analysis of beef cattle. The study population consisted of 540 Hanwoo cows: nulliparous heifers (parity 0; *n* = 236), young cows (parity 1–2; *n* = 173), mature cows (parity 3–5; *n* = 101), and older cows (parity ≥ 6; *n* = 30). Cows were retrospectively classified into the CIDR protocol (*n* = 295), GPG protocol (*n* = 124), GPPG protocol (*n* = 305), or natural estrus (*n* = 31) groups, according to the protocol they received, followed by AI. In the CIDR protocol group, cows received a GnRH injection and intravaginal CIDR insert on Day 0, a PGF_2_α injection and CIDR removal on Day 7, a second GnRH injection on Day 9, and AI on Day 10. The GPG protocol group followed the same schedule as the CIDR protocol group but used the conventional Ovsynch protocol without CIDR. The GPPG protocol group received a GnRH injection on Day 0, PGF_2_α injections on Days 5 and 6, a GnRH injection on Day 7, and AI on Day 8. Each day on the graph is divided into two boxes representing morning (8–11 AM) and afternoon (2–5 PM) treatments. Abbreviations: CIDR, progesterone-releasing device; GnRH (icon G), gonadotropin-releasing hormone; PGF_2α_ (icon P), prostaglandin F_2α_; AI (icon AI), artificial insemination.

Initiation of the Ovsynch protocol in cows during early (days 1–4) or late (days 17–21) stages of the estrous cycle may reduce pregnancy rates, whereas optimal outcomes are achieved when the protocol begins in the early to mid-luteal phase (days 5–10) of the cycle [16]. Therefore, all three synchronized ovulation protocols in this retrospective study (Figure 1) were initiated during the early to mid-luteal phase (days 5–10). For the CIDR protocol, a CIDR device (containing 1.38 g progesterone; Pfizer Animal Health, Parsippany-Troy Hills, NY, USA) was inserted intravaginally, and a GnRH analog (0.1 mg gonadorelin acetate; Fertiline, Vetoquinol, Lure, France) was administered concurrently in the morning. Seven days later, in the morning, the CIDR was removed and a PGF_2_α analog (0.5 mg cloprostenol; New Bioestrovet, Vetoquinol) was administered. Two days afterward, in the afternoon, a second GnRH analog was injected, and TAI was performed the following morning [2]. The GPG protocol followed the standard Ovsynch method. After the first GnRH injection in the morning, PGF_2_α was administered seven days later in the morning, followed two days later in the afternoon by the second GnRH injection; TAI was performed the next morning [6,12,13]. The GPPG protocol began with a first GnRH injection in the morning, followed by two PGF_2_α injections administered on the mornings of days 5 and 6. The second GnRH injection was administered the next afternoon, and TAI was conducted the following morning [17]. In all protocols, morning treatments were performed at approximately 8–11 AM, and afternoon treatments at approximately 2–5 PM. For TAI, frozen semen straws from Korean Proven Bulls (KPN bulls) supplied by the Korea Animal Improvement Association were thawed in water at 38 °C for the recommended time and loaded into an AI gun. Irrespective of estrus expression, the thawed semen was directly deposited into the uterine horn ipsilateral to the ovary bearing the DF expected to ovulate.

### 2.4. Transrectal Ultrasonography-Based Classification of DF Size and Pregnancy Diagnosis

Previous studies have demonstrated differences in pregnancy rates according to DF diameter on the day of AI. Based on these findings, this retrospective study classified DF sizes induced by various synchronized ovulation protocols. On the day of TAI, the DF diameter (mm), previously recorded by transrectal ultrasonography, was retrospectively categorized into five groups, expanding on prior classifications: very large (≥21 mm), large (17–20 mm), medium (13–16 mm), small (10–12 mm), and very small (≤9 mm). Pregnancy diagnosis was performed using ultrasonography at approximately 30 and 90 days after TAI to confirm and monitor pregnancy status. Pregnancy was confirmed by transrectal ultrasonography through identification of a gestational vesicle containing a hypoechogenic embryo within a nonechogenic area of the uterus.

### 2.5. Measurement of Serum Hormone Profiles by Enzyme-Linked Immunosorbent Assays (ELISAs) on the Day of TAI

To compare hormone profiles on the day of TAI among synchronized ovulation protocols, ELISAs were performed. As aforementioned at the inclusion criteria, blood samples used for hormone analysis were obtained from residual volumes remaining after mandatory annual blood collection. Blood samples were collected on the day of AI from selected cows, and only samples from cows that subsequently conceived after the first or second service were analyzed (*n* = 40 for CIDR, *n* = 28 for GPG, and *n* = 48 for GPPG). Immediately before AI, whole blood samples were collected via jugular venipuncture into blood coagulation tubes (BD Falcon, Franklin Lakes, NJ, USA) and transported to the laboratory under refrigerated conditions within approximately one hour to allow clotting. The supernatant was separated by centrifugation at 1000× *g* for 15 min at 4 °C. Serum samples were stored at −80 °C until hormone analysis. ELISA kits for P_4_, E_2_, and LH were obtained from Cayman Chemical Company (Ann Arbor, MI, USA). For the assay, thawed serum was mixed with enzyme immunoassay buffer, tracer, and antiserum for P_4_, E_2_, or LH, and incubated for 60 min (E_2_ and LH) or 120 min (P_4_) at room temperature (20–25 °C). Thereafter, samples in the 96-well plate were reacted with Ellman’s reagent for 60 min (P_4_ and E_2_) or with TMB substrate solution (LH) in an incubator. Absorbance was measured at 415 nm (P_4_ and E_2_) or 450 nm (LH) using a microplate reader (Epoch, Biotek, Winooski, VT, USA). Hormone concentrations in serum were calculated using a four-parameter logistic fit with free software (www.myassay.com).

### 2.6. Statistical Analysis

Statistical analysis was performed using SPSS software v.12.0 (IBM Statistical Software; IBM Corp., Armonk, NY, USA) to compare pregnancy rates and other parameters among the three synchronized ovulation protocols. Analysis of variance (ANOVA) with Duncan’s post hoc test was used to compare the mean ± standard error of the mean (SEM) for pregnancy-rate proportions calculated per batch (each batch proportion as an independent data point), since each batch is an independent experimental unit with multiple animals and more than 10 replicates per treatment; pregnancy rates by synchronization protocol and number of AIs (first, second, and third services); pregnancy rates by synchronization protocol and parity group (nulliparous heifers, young cows, mature cows, and older cows); as well as DF size at TAI and blood hormone concentrations (E_2_ and P_4_) by synchronization protocol. For ANOVA of pregnancy rates, the following conditions were applied: Although the CIDR, GPG, and GPPG protocols were performed in 295, 124, and 305 animals, respectively, data from batches containing only 1–2 cows per protocol were excluded to ensure statistical normality and homogeneity of variance. Such small batches could produce extreme values (0% or 100%), which do not satisfy the assumptions of parametric testing. Consequently, 25, 14, and 31 batch results for CIDR (275 animals), GPG (112 animals), and GPPG (248 animals), respectively, were included in the final mean comparisons by ANOVA.

Additionally, Chi-square tests were used for cross-tabulation analysis of CIDR (295 animals), GPG (124 animals), and GPPG (305 animals) protocols for the following variables: (1) pregnancy status by DF size (very large, large, medium, small, and very small); (2) follicle size distribution by synchronization protocol; and (3) pregnancy status according to whether the synchronization protocol was changed (Changed or Unchanged) for subsequent insemination after TAI failure with a previous synchronization protocol.

## 3. Results

### 3.1. Pregnancy Rates by Synchronized Ovulation Protocol

During the retrospective analysis period, a total of 793 AI results initially met inclusion criteria (1) through (9). However, 38 animals that were positive at the initial pregnancy diagnosis approximately 30 days post-TAI showed embryonic loss during the second pregnancy monitoring at approximately 75 days post-TAI and were subsequently excluded from the analysis (embryonic loss rate: 4.8%). Ultimately, 755 AI results from 540 cattle that satisfied all inclusion criteria (1) through (10) were used for the final analysis. These comprised 31 natural estrus cycles and 724 TAI following synchronized ovulation protocols. The overall pregnancy rate was 56.3% (425/755). As shown in Supplementary Figure S1, the CIDR protocol (58.3 ± 2.5%) produced a significantly higher pregnancy rate (*p* < 0.05) compared with the GPG protocol (47.5 ± 3.2%), whereas the GPPG protocol (55.5 ± 3.1%) yielded pregnancy rates statistically comparable to the CIDR protocol. Both CIDR and GPPG protocols achieved similar average pregnancy rates as breeding at natural estrus (54.8%, 17/31) whereas the GPG protocol resulted in significantly lower average pregnancy rates.

### 3.2. Pregnancy Rates by Synchronized Ovulation Protocol and Service Number

**Figure 2:**
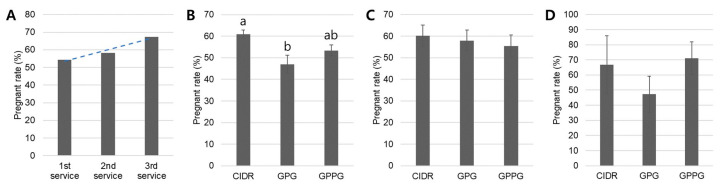
Pregnancy rates by protocol and service number. (**A**) As the number of inseminations (first, second, and third services) after synchronized ovulation increased, the mean pregnancy rates showed a gradual upward trend (a dashed line). (**B**) At the first AI, the CIDR protocol resulted in a significantly higher pregnancy rate compared with the GPG protocol, whereas the GPPG protocol produced a pregnancy rate statistically equivalent to the CIDR protocol. (**C**,**D**) When cows that failed to conceive after the first AI were resynchronized, no significant differences (*p* > 0.05) in pregnancy rates among protocols were observed during the second and third AI attempts. Significant differences (*p* < 0.05) are indicated by different letters above the bars. Graphs are presented as mean (**A**) or mean ± standard error of the mean (SEM; **B**–**D**). Sample sizes: 1st AI—CIDR (214/14), GPG (82/7), GPPG (166/16); 2nd AI—CIDR (52/8), GPG (19/4), GPPG (64/10); 3rd AI—CIDR (9/3), GPG (11/3), GPPG (18/5), where numbers indicate total animals/independent trials.

Pregnancy rates by synchronization protocol and service number are shown in Figure 2. In this retrospective analysis, the average pregnancy rates progressively increased across services, with rates of 54.4% (295/542), 58.3% (95/163), and 67.4% (31/46) for the first, second, and third services, respectively (Figure 2A). A total of four cows received a fourth service; however, none conceived (pregnancy rate: 0%). This progressive decrease in the number of cows advancing from first to subsequent services represents a limitation of retrospective studies, as strict experimental control over animal retention and repeat insemination is not feasible under practical farm management conditions. Retrospective analysis of pregnancy rates by service number showed a significant increase (*p* < 0.05) in the CIDR protocol (61.0 ± 1.8%) compared with the GPG protocol (47.0 ± 4.2%) at the first service (Figure 2B). However, no significant differences were observed among synchronization protocols during the second and third services (Figure 2C,D). Furthermore, in Table 1, the pregnancy rate among cows whose synchronization protocol was changed was 32/85 (37.6%), while the rate among cows with no protocol change was 47/128 (36.7%). A Chi-square test confirmed no significant difference between these groups (χ^2^ = 0.019, *p* = 0.891), indicating that altering the synchronization protocol did not affect overall pregnancy success.

Taken together, these findings indicate that the CIDR protocol is more effective during the first AI; in subsequent inseminations, pregnancy outcomes do not substantially differ among protocols. This may be explained by the fact that cows more responsive to the CIDR protocol conceive early and are therefore excluded from the re-insemination group, leaving no discernible differences in protocol responsiveness among cows requiring additional services.

### 3.3. Pregnancy Rates by Synchronized Ovulation Protocol and Parity

**Figure 3:**
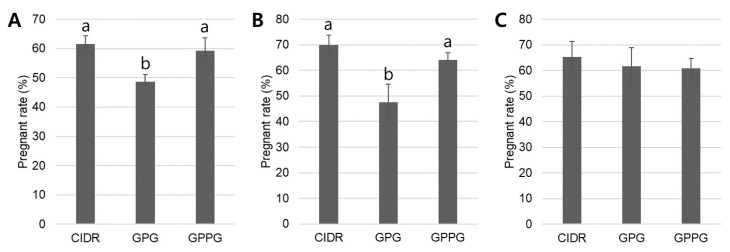
Pregnancy rates by protocol and parity. (**A**) In nulliparous heifers, pregnancy rates were significantly higher in the CIDR and GPPG groups compared with the GPG group. (**B**) In young cows, pregnancy rates were also significantly higher in the CIDR and GPPG groups than in the GPG group. (**C**) In mature cows, protocol selection did not significantly (*p* > 0.05) affect pregnancy outcomes. Significant differences (*p* < 0.05) are indicated by different letters above the bars. Graphs are presented as mean ± SEM. Sample sizes: nulliparous heifers—CIDR (127/18), GPG (72/9), GPPG (121/15); young cows—CIDR (66/13), GPG (23/4), GPPG (89/16); mature cows—CIDR (51/9), GPG (15/3), GPPG (22/4), where numbers indicate total animals/independent trials.

In retrospective analysis of pregnancy rates across the four parity groups, pregnancy rates in nulliparous heifers and young cows were more strongly influenced by synchronization protocol compared with rates in mature and older cows. In nulliparous heifers (Figure 3A), the CIDR (61.6 ± 2.8%) and GPPG (59.3 ± 4.3%) protocols resulted in significantly higher pregnancy rates (*p* < 0.05) than the GPG protocol (48.6 ± 2.6%). Consistent with nulliparous heifers, in young cows (Figure 3B), both the CIDR (70.0 ± 3.9%) and GPPG (64.1 ± 2.9%) protocols produced significantly higher pregnancy rates (*p* < 0.05) compared with the GPG protocol (47.5 ± 7.2%). In contrast, protocol selection did not significantly affect pregnancy outcomes in mature cows (Figure 3C). For older cows, statistical analysis was not performed because of insufficient frequency in each batch. However, mean pregnancy rates were 44.4% for CIDR, 46.1% for GPG, and 50.0% for GPPG, showing a trend similar to that observed in mature cows.

### 3.4. Pregnancy Rates by Synchronized Ovulation Protocol and DF Size

Retrospective analysis first examined the relationship between DF size on the day of AI and pregnancy rates. As shown in Table 2, pregnancy rates following each synchronization protocol varied considerably by DF size, ranging from 28.6% to 66.2% (excluding the very large DF group with frequency <5). Chi-square analysis revealed that across all synchronization protocols combined, medium-sized follicles (13–16 mm) were associated with the highest pregnancy rates compared to other follicle size categories (64.5%; χ^2^ = 25.88, *p* < 0.01). The distribution of DF size categories by synchronized ovulation protocol is shown in Supplementary Figure S2. Although the mean DF size across all synchronization protocols and natural estrus fell within the medium range associated with the highest pregnancy rates, the CIDR protocol (14.1 ± 0.2 mm) induced significantly larger DFs (*p* < 0.05) than the other protocols (GPG: 13.3 ± 0.4 mm; GPPG: 13.0 ± 0.2 mm). As shown in Table 3, Chi-squared analysis demonstrated that the CIDR protocol increased the frequency of medium-sized follicles associated with the best pregnancy outcomes, with medium-sized follicles (13–16 mm) comprising 50.2% of follicles in the CIDR group compared to 35.5% in the GPG group and 35.1% in the GPPG group. In contrast, the GPG and GPPG protocols showed a higher frequency of very small follicles (6–9 mm), which correspond to the lower pregnancy rate (37.1%) observed in Table 2. The proportion of very small follicles was 2.4% in the CIDR group, while it was 17.7% in the GPG group and 19.7% in the GPPG group (χ^2^ = 51.04, *p* < 0.01).

### 3.5. Changes in Reproductive Hormones According to Synchronized Ovulation

**Figure 4:**
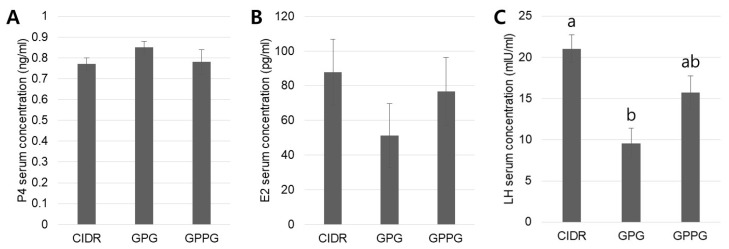
Reproductive hormone levels by protocol. (**A**) On the day of AI, the GPG protocol group showed a tendency toward higher blood P_4_ concentrations compared with the other two groups (*p* > 0.05). (**B**) E_2_ concentrations tended to be higher in the CIDR and GPPG groups compared with the GPG group (*p* > 0.05). (**C**) The CIDR protocol induced significantly higher blood LH concentrations compared with the GPG protocol. Significant differences (*p* < 0.05) are indicated by different letters above the bars. Graphs are presented as mean ± SEM. Abbreviations: P_4_, progesterone; E_2_, estradiol; LH, luteinizing hormone. Sample sizes: *n* = 40 for CIDR, *n* = 28 for GPG, and *n* = 48 for GPPG.

Serum samples collected on the day of AI were analyzed for concentrations of major reproductive hormones in cows that conceived after the first and second services (Figure 4). For P_4_, no significant differences were observed among the three protocols, although the GPG protocol showed a slight tendency toward higher concentrations compared with the other two groups. E_2_ levels tended to be higher in the CIDR protocol than in the GPG protocol, although this difference was not statistically significant. This tendency may be associated with the larger DF observed in the CIDR group on the day of AI. For LH concentrations, the pattern was similar to that of E_2_, but the difference between the CIDR and GPG protocols was significant (*p* < 0.05; CIDR: 21.05 ± 1.70 mIU/mL, GPG: 9.57 ± 1.82 mIU/mL). This finding may reflect insufficient positive feedback of E_2_ for induction of the LH surge in the GPG group.

## 4. Discussion

After synchronized ovulation, TAI provides substantial benefits to cattle production by reducing the interval from calving to subsequent conception, enhancing fertility in cows with anestrous ovaries, and minimizing the need for estrus detection, which remains a major obstacle in large herds and grazing systems. Consequently, hormone-based synchronized ovulation protocols have been commercially utilized for decades; ongoing refinement continues to improve reproductive efficiency and conception rates in cattle [6,18]. Among available synchronized ovulation strategies, the CIDR and GPG protocols represent two fundamental approaches, each based on distinct endocrine mechanisms. The CIDR protocol, developed in the 1990s, employs an intravaginal device that releases P_4_, temporarily simulating the luteal phase. Elevated circulating P_4_ suppresses ovulation and follicular development; subsequent device removal combined with PGF_2_α administration induces regression of the CL. The resulting decline in P_4_ concentrations triggers synchronized estrus and ovulation within a controlled time frame. This method ensures that even anestrous or non-cycling females receive sufficient P_4_ priming, thereby enhancing synchrony and fertility [2,5,12]. CIDR protocols have demonstrated pregnancy rates of 47.0–67.2% in heifers and 54.0–63.3% in parous cows [3,6,11,18,19,20]. Consistent with these reports, the present retrospective study also showed high pregnancy rates with the CIDR protocol: 61.6% in heifers and 65.3–70.0% in young and mature cows. These high and consistent pregnancy rates observed in both heifers and parous cows confirm that the CIDR protocol among our synchronized ovulation protocols can effectively achieve the study’s objective of improving reproductive efficiency.

In the GPG protocol, the first GnRH injection initiates ovulation or luteinization of the dominant follicle, resetting the follicular wave. This is followed by administration of PGF_2α_, which induces luteolysis and decreases endogenous P_4_ levels. Subsequently, the second GnRH injection triggers ovulation of the newly developed dominant follicle, allowing timed artificial insemination without the need for estrus detection [2,5]. The hormonal control achieved here is based on precise exogenous regulation of the hypothalamic–pituitary–ovarian axis, a strategy that is particularly effective in cyclic females [12]. As a result, the GPG protocol has been widely adopted in cattle herds and typically produces consistent pregnancy outcomes. Published pregnancy rates include 35.1–39.1% in heifers, 37.8–51.2% in parous cows, 42.4–44.3% in beef cattle, and 37.8–51.2% in dairy cattle; these figures align closely with the retrospective findings of this study, which reported 48.6% in nulliparous heifers and 47.5–61.7% in parous cows [6,8,12,13,14,21]. When the GPG protocol is applied to postpartum anestrous cows, a pregnancy rate of 33.3–50.0% is achieved, which, although lower than the rates observed in cyclic controls (50.0–83.3%), still represents a marked improvement over untreated controls [22,23]. Despite these positive results, the pregnancy rates associated with the GPG protocol are generally inferior to those obtained with the CIDR protocol. Direct comparison studies indicate consistently higher pregnancy rates with CIDR than GPG in parous beef cattle (CIDR: 54.0–54.6%; GPG: 43.0–44.3%) [6,12], as well as in postpartum anestrous cows (CIDR: 60.0–66.7%; GPG: 33.3–50.0%) [22,23]. The enhanced performance of CIDR is likely attributable to more uniform luteal support and stricter control of follicular development, whereas GPG’s dependence on endogenous follicular status increases variability in ovulatory response. Nevertheless, as a retrospective analysis, our study cannot account for all confounding factors, such as unmeasured variable (seasonality) or full management control. These inherent limitations must be considered when interpreting comparative results. However, consistent with previous reports, our current analysis also demonstrated that the CIDR protocol produced significantly higher pregnancy rates than GPG in the overall herd (58.3% vs. 47.5%), a difference that was especially notable in nulliparous heifers (CIDR: 61.6% vs. GPG: 48.6%) and young cows (CIDR: 70.0% vs. GPG: 47.5%).

The GPPG protocol is an advanced form of TAI that addresses the limitations of the conventional Ovsynch program through strategic hormonal manipulation and optimized timing intervals. Its principal advantage lies in substantially improving pregnancy rates by reducing the period of follicular dominance from 10 days to 8 days, thus preventing ovulation of aged or compromised oocytes [17]. The endocrinological basis involves administering two doses of PGF_2_α at 24 h intervals on days 5 and 6 after the initial GnRH injection. This strategy ensures complete luteolysis of both natural and newly formed CLs, given that a single PGF_2_α treatment is ineffective against CLs less than 5 days old [24]. This hormonal coordination maintains appropriate P_4_ concentrations during follicular development and guarantees the presence of a suitably sized DF at the time of the final GnRH injection, ultimately resulting in improved synchronization and pregnancy outcomes compared with the standard 7-day protocol. Previous studies have reported that the GPPG protocol improves reproductive performance compared with the GPG protocol, yielding higher luteolysis rates on the day of AI (GPPG: 96.3% vs. GPG: 91.5%), estrus synchronization rates (GPPG: 81.0% vs. GPG: 74.3%), and pregnancy rates (GPPG: 37.9% vs. GPG: 30.9%) [17]. Consistent with these findings, the present retrospective study demonstrated that the GPPG protocol produced higher pregnancy rates than the GPG protocol in nulliparous heifers and young cows. Moreover, its outcomes were comparable to those of the CIDR protocol: overall herd (CIDR: 58.3%; GPG: 47.5%; GPPG: 55.5%), nulliparous heifers (CIDR: 61.6%; GPG: 48.6%; GPPG: 59.3%), and young cows (CIDR: 70.0%; GPG: 47.5%; GPPG: 64.1%).

The cows in this retrospective study were classified into four parity groups, and pregnancy rates were analyzed according to synchronized ovulation protocol [25,26]. Generally, nulliparous heifers (parity 0) have high energy requirements because pregnancy progresses simultaneously with growth. They are also known to exhibit weaker estrus expression and require more time to reach first ovulation compared with mature cows. Young cows (parity 1–2) are characterized by nearly completed growth, enhanced reproductive capacity, and relatively high pregnancy rates. Mature cows (parity 3–5) reach peak milk production in dairy cattle but begin to experience a decline in reproductive performance due to increased metabolic stress. Older cows (parity ≥ 6) are characterized by slower body condition recovery, higher disease incidence, and an overall decline in reproductive capacity. In this retrospective analysis, pregnancy rates were highest in young cows and lowest in older cows. Importantly, the CIDR and GPPG protocols demonstrated significantly greater effectiveness than the GPG protocol in nulliparous heifers and young cows (CIDR: 61.6–70.0%; GPG: 47.5–48.6%; GPPG: 59.3–64.1%), whereas protocol selection had little effect on pregnancy outcomes in mature and older cows. Although relatively few studies have subdivided parity to this extent and directly compared pregnancy rates across protocols (CIDR vs. GPG vs. GPPG), a comparison of two studies confirmed a tendency for the CIDR protocol to increase pregnancy rates relative to the GPG protocol in young cows (CIDR: 44.0–55.1%; GPG: 39.3–50.0%) [6,27].

The progressive increase in pregnancy rates with successive services observed in this retrospective study (54.4% for the first, 58.3% for the second, and 67.4% for the third) aligns with previous findings demonstrating improved fertility outcomes in repeat-bred animals. The significantly higher first-service pregnancy rate achieved with the CIDR protocol (61.0%) compared with the GPG protocol (47.0%) supports the beneficial effect of P_4_ supplementation in synchronized ovulation protocols. This result is consistent with a previous study reporting that CIDR-based protocols yielded pregnancy rates of 58% compared with 53% for protocols lacking P_4_ supplementation [6,28]. The superior performance of CIDR at first service can be attributed to the P_4_ priming effect, which enhances follicular development and ovulation synchrony, particularly in anestrous or irregularly cycling cows [22]. The absence of significant differences among protocols in subsequent services (second and third) suggests that the initial hormonal intervention establishes a more favorable reproductive environment that persists beyond the first breeding attempt. This interpretation is supported by previous studies showing that progestin-based protocols effectively induce cyclicity in previously anestrous animals, leading to improved subsequent reproductive performance [6,28]. Moreover, the lack of significant differences when protocols were switched in later services (χ^2^ = 0.019, *p* = 0.891) indicates that protocol selection for repeat services may be less critical once normal cyclicity is established, offering practical flexibility for breeding management. However, our interpretation that protocol choice becomes irrelevant after the first AI should be considered with caution. Cows requiring multiple services may represent a biologically distinct subgroup with altered reproductive physiology. The apparent lack of protocol differences in subsequent services may reflect biological selection in our retrospective design, where responsive cows conceive early and are excluded from repeat analyses.

The relationship between DF size at AI and pregnancy rates observed in this retrospective study is consistent with numerous reports identifying optimal follicle diameter ranges for successful conception. The finding that medium-sized follicles (13–16 mm) achieved the highest pregnancy rates (64.5%) aligns with prior studies demonstrating maximum pregnancy rates of 56.3% at a follicle size of 14.6 mm and optimal pregnancy outcomes in dairy cows with follicles between 13.5 and 17.5 mm [29,30]. The significantly larger DF sizes induced by the CIDR protocol (14.1 mm) relative to GnRH-based protocols (GPG: 13.3 mm; GPPG: 13.0 mm) may explain the superior pregnancy outcomes associated with P_4_–E_2_-based synchronization. This observation is supported by a previous report indicating that adjustment of AI timing according to follicle diameter can improve fertility, particularly by allowing smaller follicles additional time to mature [31]. Furthermore, the higher frequency of very small follicles (<10 mm) observed in GnRH-based protocols (GPG: 17.7%; GPPG: 19.7%) compared with CIDR (2.4%) is concerning, given that follicles smaller than 11 mm have been linked to increased late embryonic mortality and reduced pregnancy rates due to inadequate CL function and insufficient P_4_ production [29,32]. The ability of the CIDR protocol to consistently induce medium-sized follicles may be explained by its extended proestrus period and more controlled follicular development relative to the compressed timeline of GnRH-based protocols.

In this study, among the three synchronized ovulation protocols, the CIDR protocol achieved the highest pregnancy rate (58.3%) and was characterized at the time of AI by relatively lower P_4_ concentrations, relatively higher E_2_ levels, and a significantly elevated LH concentration. The superior performance of the CIDR protocol may be attributed to several interconnected mechanisms related to P_4_ supplementation and its effects on follicular development and ovulation synchronization. During the synchronization period, exogenous P_4_ provided by the CIDR device establishes an optimal hormonal environment that promotes more complete luteal regression compared with protocols lacking external P_4_ support [12,33]. This improved luteal regression is reflected in the lower P_4_ concentrations observed at the time of AI, which is advantageous for ovulation synchronization because elevated P_4_ can suppress GnRH-induced LH secretion and disrupt the timing of ovulation [7,23]. The larger DFs observed in the CIDR group are likely associated with increased E_2_ production capacity [15]. The significantly higher LH concentration measured in the CIDR group (21.05 mIU/mL) compared with the GPG group (9.57 mIU/mL) suggests that elevated E_2_ enhanced GnRH-induced LH secretion, resulting in a stronger preovulatory LH surge through positive feedback mechanisms [34]. In contrast, the GPG protocol showed the lowest pregnancy rate (47.5%), accompanied by relatively high P_4_, relatively low E_2_, and significantly reduced LH levels, indicating incomplete luteal regression, a known limitation of the standard Ovsynch protocol [17,24]. The elevated P_4_ environment in the GPG group may have suppressed GnRH responsiveness and reduced E_2_ production, thereby impairing the positive feedback required for an optimal LH surge [34]. The GPPG protocol produced intermediate results (55.5% pregnancy rate) with a hormonal profile similar to that of the CIDR protocol. This outcome suggests that double PGF_2_α administration improved luteal regression and created a more favorable hormonal environment for ovulation synchronization [17,24]. While our results suggest that the CIDR protocol’s superior performance is primarily due to hormonal mechanisms, we acknowledge that other factors, including management differences and technician experience, may contribute to the observed pregnancy rate variations. Furthermore, our hormonal analysis was limited to pregnant animals, which may introduce survivorship bias. However, our retrospective design inherently limits the ability to control for several confounders, and this analytical approach was deliberately chosen to elucidate at least one positive reproductive endocrinological mechanism underlying the superior performance of the CIDR protocol; by analyzing hormone profiles from successfully pregnant animals, we sought to identify the optimal hormonal environment associated with reproductive success under each synchronization protocol, providing mechanistic insight into why certain protocols achieve higher pregnancy rates. While we acknowledge that multiple factors can influence pregnancy rates in cattle operations, this focused hormonal analysis provides valuable mechanistic evidence that complements the observed reproductive outcomes.

## 5. Conclusions

This retrospective analysis demonstrated that the CIDR protocol achieved superior pregnancy rates compared with the GPG protocol, whereas the GPPG protocol produced intermediate outcomes that were statistically comparable to CIDR. The benefits of synchronization protocols were most evident in younger animals (nulliparous heifers and young cows) and at the first service; protocol selection was less critical in older, more reproductively experienced animals and in subsequent services. These findings provide important insights for the development of optimized reproductive management strategies tailored to native beef cattle operations, addressing the previously limited knowledge of synchronized ovulation efficacy in this genetically distinct population under regional climatic and management conditions.

## Figures and Tables

**Table 1:** Cross-tabulation of pregnancy status by synchronization protocol change.

	Protocol Changed	Protocol Unchanged	χ^2^
Pregnant	32 (37.6)	47 (36.7)	0.019 ^ns^
Non-pregnant	53 (62.4)	81 (63.3)	
Total	85 (100.0)	128 (100.0)	

Data are presented as number of animal (% of total). Statistical analysis: Chi-square test; ^ns^ *p* = 0.891.

**Table 2:** Pregnancy rate and dominant follicle size: Cross-tabulation analysis.

Groups	Very Small (6–9 mm)	Small (10–12 mm)	Medium (13–16 mm)	Large (17–20 mm)	Very Large (<21 mm)	χ^2^
CIDR	28.6% (2/7)	58.8% (50/85)	66.2% (98/148)	51.0% (26/51)	0.0% (0/4)	-
GPG	31.8% (7/22)	46.9% (15/32)	59.1% (26/44)	43.5% (10/23)	66.7% (2/3)	
GPPG	40.0% (24/60)	61.2% (52/85)	64.5% (69/107)	51.0% (25/49)	50.0% (2/4)	
Total	37.1% (33/89)	57.9% (117/202)	64.5% (193/299)	49.6% (61/123)	36.4% (4/11)	25.88 *

Data are presented as pregnancy rate (number of pregnant cows/total number of cows) of each dominant follicle size group. Statistical analysis: Chi-square test; * *p* < 0.05.

**Table 3:** Dominant follicle size and synchronized ovulation protocol: Cross-tabulation analysis.

Groups	Very Small (6–9 mm)	Small (10–12 mm)	Medium (13–16 mm)	Large (17–20 mm)	Very Large (<21 mm)	χ^2^
CIDR	7 (2.4)	85 (28.8)	148 (50.2)	51 (17.3)	4 (1.4)	51.04 *
GPG	22 (17.7)	32 (25.8)	44 (35.5)	23 (18.5)	3 (2.4)	
GPPG	60 (19.7)	85 (27.9)	107 (35.1)	49 (16.1)	4 (1.3)	

Data are presented as the number of animals per synchronization protocol (% of the total). Statistical analysis: Chi-square test; * *p* < 0.05

## Data Availability

The original contributions presented in this study are included in the article/Supplementary Material. Further inquiries can be directed to the corresponding author.

## Supplementary Materials

The following supporting information can be downloaded at: https://www.mdpi.com/article/10.3390/vetsci12101001/s1, Figure S1. Pregnancy rates according to synchronized ovulation protocol in retrospective analysis, Figure S2. Dominant follicle size according to synchronized ovulation protocol in retrospective analysis.

## Abbreviations

The following abbreviations are used in this manuscript:

CIDR	Progesterone-releasing devices
CL	Corpus luteum
DF	Dominant follicle
E_2_	Estradiol
FSH	Follicle-stimulating hormone
GnRH	Gonadotropin-releasing hormone
LH	Luteinizing hormone
P_4_	Progesterone
PGF2α	Prostaglandin F2α
TAI	Timed artificial insemination
