# Supplementary material for: Effects of Synchronized Ovulation Protocols on Reproductive Performance of Beef Cattle in Korea: A Retrospective Study of 755 Cases
Source: Vet Sci. 2025 Oct 16;12(10):1001. doi: 10.3390/vetsci12101001 (PMC12567625; doi:10.3390/vetsci12101001)
Supplement: Supplementary file 1 [file vetsci-12-01001-s001.zip › vetsci-3880023-supplementary.pdf]

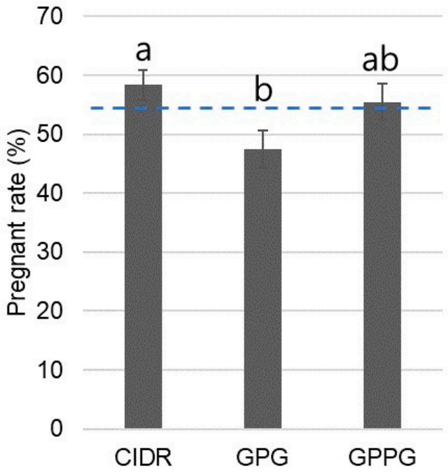

Supplementary Figure S1. Pregnancy rates according to synchronized ovulation protocol. The CIDR protocol achieved a significantly higher pregnancy rate compared with the GPG protocol. Both CIDR and GPPG protocols demonstrated higher average pregnancy rates than natural estrus breeding (indicated by the blue dashed line), whereas the GPG protocol showed the opposite trend. Significant differences ($p<0.05$) are indicated by different letters above the bars. Graphs are presented as mean±SEM.

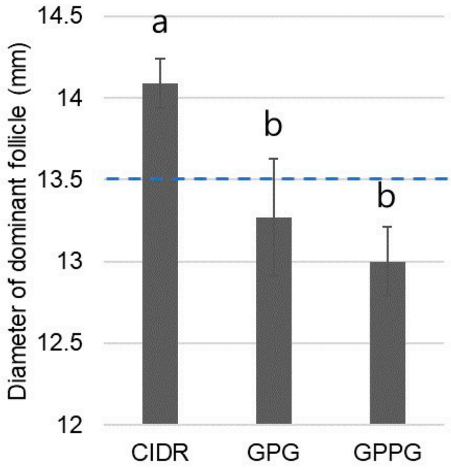

Supplementary Figure S2. Dominant follicle size according to synchronized ovulation protocol. The CIDR protocol induced larger dominant follicle sizes compared with the other groups. Significant differences ($p<0.05$) are indicated by different letters above the bars. Graphs are presented as mean±SEM.
